# Genome-wide DNA methylation profiling reveals candidate biomarkers and probable molecular mechanism of metabolic syndrome

**DOI:** 10.1016/j.gendis.2021.12.010

**Published:** 2022-01-11

**Authors:** Su-Jin Baek, Hyo-Jeong Ban, Sang-Min Park, Soo Yeon Kim, Siwoo Lee, Hee-Jeong Jin

**Affiliations:** KM Data Division, Korea Institute of Oriental Medicine, 1672 Yuseong-daero, Yuseong-gu, Daejeon 34054, Republic of Korea

Although aberrant DNA methylation changes are significantly associated with the pathogenesis of many diseases, little is known about the molecular mechanisms underlying interactions between DNA methylation and gene expression in metabolic syndrome (MetS). The aim of this study was to identify biomarkers and molecular mechanisms regulated by DNA methylation in MetS. We profiled genome-wide DNA methylation using the Infinium MethylationEPIC BeadChIP and characterized the transcriptome by RNA sequencing in our study population comprised of subjects with MetS (*n* = 11) and controls (*n* = 9). In total, 13,707 significantly differentially methylated probes (DMPs) were identified in subjects with MetS, most in the promoter and coding regions. Among them, 47 DMPs were significantly correlated with the expression of 36 corresponding genes, which were enriched in ‘insulin resistance’, ‘insulin signaling pathway’, and the ‘apelin signaling pathway’. Among these MetS-associated genes, validation of the most discriminating gene via ROC curve analysis, *GFPT2*, showed significant hypermethylation/downregulated expression in subjects with MetS compared to that in normal controls via bisulfite amplicon sequencing (BSAS) and quantitative real-time PCR (qRT-PCR). Our findings demonstrated altered DNA methylation in subjects with MetS, suggesting that *GFPT2* hypermethylation might be a promising epigenetic biomarker and emphasizing the role of aberrant GFPT2 expression in MetS pathogenesis.

The prevalence of MetS is gradually increasing and ranges from 10% to 40% worldwide.^1^ MetS is an important risk factor for cardiovascular disease and diabetes mellitus.^2^ The molecular mechanism underlying MetS is multifactorial and complex, which is compounded by the lack of effective biomarkers and knowledge of mechanical pathways driven by DNA methylation. Abnormal DNA methylation in promoters plays an important role in the regulation of gene activity and has been studied as a biomarker for various diseases. Methylation of CpG islands (CGIs; high-density CpG regions) and regions upstream of transcription start sites (TSS) are widely known mechanisms associated with gene expression regulation. Here, we correlated MethylationEPIC BeadChIP data with RNA-seq data based on samples obtained from the same subjects with MetS to elucidate the molecular mechanism underlying MetS and search for novel epigenetic biomarkers for this disorder. Toward this, 32 participants, including 18 subjects with MetS and 14 controls, were enrolled in this study. The patient characteristics are listed in Table S1 and a flow chart of the overall study design is depicted in Figure S1. To identify DMPs in MetS patients *vs.* normal controls, we profiled the DNA methylome of 11 MetS and nine control samples using the Illumina Human MethylationEPIC BeadChIP. Approximately 0.96% (3509/363,651) of hypermethylated and 2.8% (10,198/363,651) of hypomethylated DMPs were selected from a total of 363,651 probes (*P* < 0.05; Fig. 1A; Table S2). Hypermethylated regions were more frequently observed in the gene body and intergenic region (IGR) than in other genomic regions, whereas hypomethylated regions were more frequently observed in the TSS200 and TSS1500 regions (Fig. 1B, left panel). In CpG regions, hypermethylated probes were more frequently observed in open sea, whereas hypomethylated probes were more frequently observed in islands and shores (Fig. 1B, right panel). We annotated the genes with the closest DMPs, including 2021 genes with hypermethylated probes and 5220 genes with hypomethylated probes using Infinium MethylationEPIC BeadChIP annotation (Fig. 1C). To decipher the biological involvement of genes with DMPs, we performed gene set enrichment analysis (Table S3). Hypermethylated genes were enriched in functional terms such as ‘insulin secretion’ and ‘glutamatergic synapse’. Hypomethylated genes were enriched in terms such as ‘Wnt signaling pathway’, ‘MAPK pathway’, and ‘Hippo pathway’.

**Figure 1:**
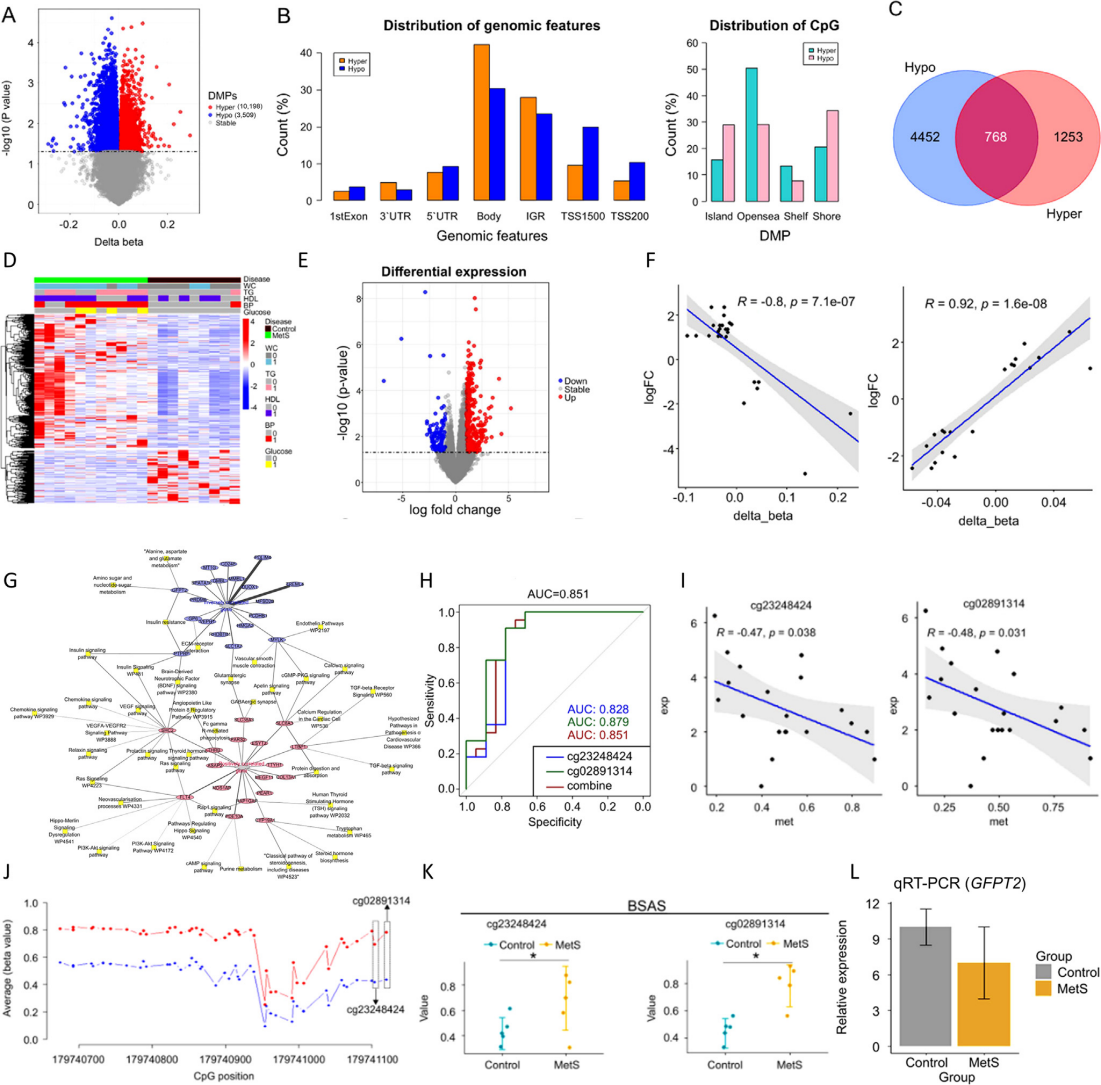
Epigenetic targets and mechanisms regulated by DNA methylation in subjects with MetS, compared to those in controls, and assessment of putative epigenetic targets for MetS diagnosis. **(A)** Volcano plot of differentially methylated probes (DMPs) in MetS *vs.* control. **(B)** Distribution of DMPs in genomic features and CpG regions. The left panel illustrates the distribution of DMPs in genomic regions (1st exon, 3′ UTR, 5′ UTR, body, intergenic region (IGR), TSS1500, and TSS200), whereas the right panel illustrates the distribution of DMPs relative to CpG island groups (island, shore, shelf, and open sea). TSS200/1500 are defined as 200/1500 bp regions upstream from the transcription start site. Island groups are defined as CG dinucleotide-rich regions. Shores are defined as 2 Kb regions upstream/downstream from the ends of CpG islands. A shelf is defined as another 2 Kb region upstream/downstream of the farthest upstream/downstream limits of the CpG shores. Open seas are defined as CpG sites in the genome that do not have a specific designation. **(C)** Venn diagram of DMPs. The blue/red circles represent the numbers of hyper/hypomethylated genes. **(D)** Heat map of DEGs in MetS *vs.* control. The upper panel illustrates subject risk factors (0, Control; 1, risk determinants). **(E)** Volcano plot of differentially expressed genes (DEGs) in MetS *vs.* control. **(F)** Correlation scatter plot between DNA methylation status and gene expression. The x-axis represents delta-beta values of DNA methylation and the y-axis represents fold-changes in gene expression in MetS *vs.* control. **(G)** Integrated functional network for metabolic syndrome (MetS) correlated based on DNA methylation and gene expression. The blue/red circles denote negatively/positively correlated genes, and yellow squares denote related functional terms. The thickness of the line represents the weight of the *P*-value. **(H)** Diagnostic power of the two CpG probes in *GFPT2* using receiver operating characteristic (ROC) analysis for cg02891314, cg23248424, and combined cg02891314 and cg23248424. **(I)** Correlation scatter plot for cg23248424 and cg02891314. **(J)** Validation of DNA methylation values for two CpG probes using bisulfite amplicon sequencing. The red/blue lines represent metabolic syndrome (MetS)/control, and the red/blue dots represent CpG sites within 497 bp for MetS/control (*n* = 10). **(K)** Scatter box plot for the two candidate CpG probes. The blue and yellow dots indicate control (*n* = 5) and MetS (*n* = 5) samples, respectively. **(L)** Validation of *GFPT2* expression using qRT-PCR. We performed relative quantification using the 2^−ΔΔCt^ method with *GAPDH* as the internal control, and the data are expressed as the relative fold change (FC). Statistical significance between two groups was analyzed using two-tailed *t*-tests. The blue and yellow boxes represent the control (*n* = 5) and MetS (*n* = 4) groups, respectively. ∗*P* < 0.05.

To identify differentially expressed genes (DEGs) and key pathways involved in MetS, we performed gene expression profiling using total RNA-seq data of patients with MetS (*n* = 11) and healthy controls (*n* = 9; Fig. 1D). Among 517 DEGs, expression levels of 148 DEGs were downregulated, while those of 369 DEGs were upregulated in MetS compared to expression levels in controls (|logFC| < 2 and *P* < 0.05; Fig. 1E and Table S4). DEGs were enriched in functions related to ‘atrial fibrillation’, ‘coronary heart disease’, ‘coronary artery disease’, ‘coronary arteriosclerosis’, and ‘atherosclerosis’ using DisGeNET gene sets (Table S5). To identify MetS-associated genes regulated by DNA methylation, we further performed a correlation analysis between DNA methylation and gene expression. In total, 16 hypermethylated and 31 hypomethylated probes were significantly correlated with the expression of their corresponding genes (*P* < 0.05; Fig. 1F and Table S6). These 36 genes were enriched in gene ontology (GO) functions and biological pathways pertaining to ‘insulin resistance’ (*P* = 0.016; *GFPT2*, *PTPRF*), ’insulin signaling pathway’ (*P* = 0.03; *SHC2*, *PTPRF*), and ‘apelin signaling pathway’ (*P* = 0.02; *SLC8A3*, *MYLK*), indicating that changes in the methylation status of the candidate genes play important roles in regulating insulin-related functions. We subsequently performed a functional integrated network analysis of DNA methylation and gene expression using the 36 MetS-associated genes and the 25 functional pathways detected in the functional enrichment analysis described previously herein (Table S7). Fig. 1G illustrates the predicted pathways and genes related to epigenetically regulated MetS-associated genes and pathways. ROC curve analysis was performed to predict the diagnostic performance of candidate biomarkers in MetS. Among the epigenetically regulated MetS-associated genes, *GFPT2* was prominent, with area under curve (AUC) values of 0.828 for cg23248424 and 0.879 for cg02891314. The combined AUC value for cg232484242 and cg02891314 was 0.851 (Fig. 1H). We then validated the novel, epigenetically regulated MetS-associated genes, focusing on *GFPT2*, which possessed high predictive power among the identified MetS-associated genes via ROC analysis. *GFPT2*, known to encode a glycosylate protein and a member of the insulin signaling pathway, was one of the genes associated with a negative correlation between DNA methylation and gene expression (Fig. 1I). *GFPT2* encodes the first and rate-limiting enzyme glutamine-fructose-6-phosphate transaminase 2,^3^ and reportedly regulates the hexosamine biosynthetic pathway, thereby contributing to susceptibility to T2DM and diabetic nephropathy.^4^ We first performed BSAS to measure the methylation levels of *GFPT2* in MetS (*n* = 5) and control (*n* = 5) samples (Fig. 1J, K, and Table S8). We also performed qRT-PCR to measure the expression levels of *GFPT2* in MetS (*n* = 4) compared to those in control (*n* = 5) samples (Fig. 1L and Table S9). The BSAS results indicated that two probes in *GFPT2* had significant hypermethylation in MetS samples compared to levels in control samples, specifically cg23248424 (*P* = 0.046) and cg02891314 (*P* = 0.014). Both of these have been reported as candidate probes to predict adiposity outcomes using cord blood,^5^ but not as markers to directly predict metabolic disease. The qRT-PCR results demonstrated significantly decreased expression of *GFPT2* in MetS samples (*P* = 0.044). These results indicated that changes in hypermethylation at *GFPT2* might contribute to the downregulation of *GFPT2* in subjects with MetS, as compared to levels in controls.

The present study aimed to investigate the molecular mechanisms and biomarkers of DNA methylation in subjects with MetS compared to those in controls. In total, 47 probes and 36 genes were identified through correlation analysis. Among the MetS-associated genes, two CpG sites of *GFPT2*, identified based on high AUC values and validated by qRT-PCR and BSAS, have the potential to be used for MetS diagnosis. The MetS-associated genes identified in the current study might play important roles in regulating the mechanisms underlying MetS, especially insulin-related pathways, and could provide markers for the diagnosis and treatment of MetS.

## Conflict of interests

The authors declare no conflict of interests.

## Funding

This study was supported by the “Development of Korean Medicine Original Technology for Preventive Treatment based on Integrative Big Data” grant from the 10.13039/501100003718Korea Institute of Oriental Medicine (No. KSN2023120).
