# Supplementary material for: Genome-wide DNA methylation profiling reveals candidate biomarkers and probable molecular mechanism of metabolic syndrome
Source: Genes Dis. 2022 Jan 11;9(4):833–6. doi: 10.1016/j.gendis.2021.12.010 (PMC9170599; doi:10.1016/j.gendis.2021.12.010)
Supplement: Multimedia component 1 [file mmc1.docx]

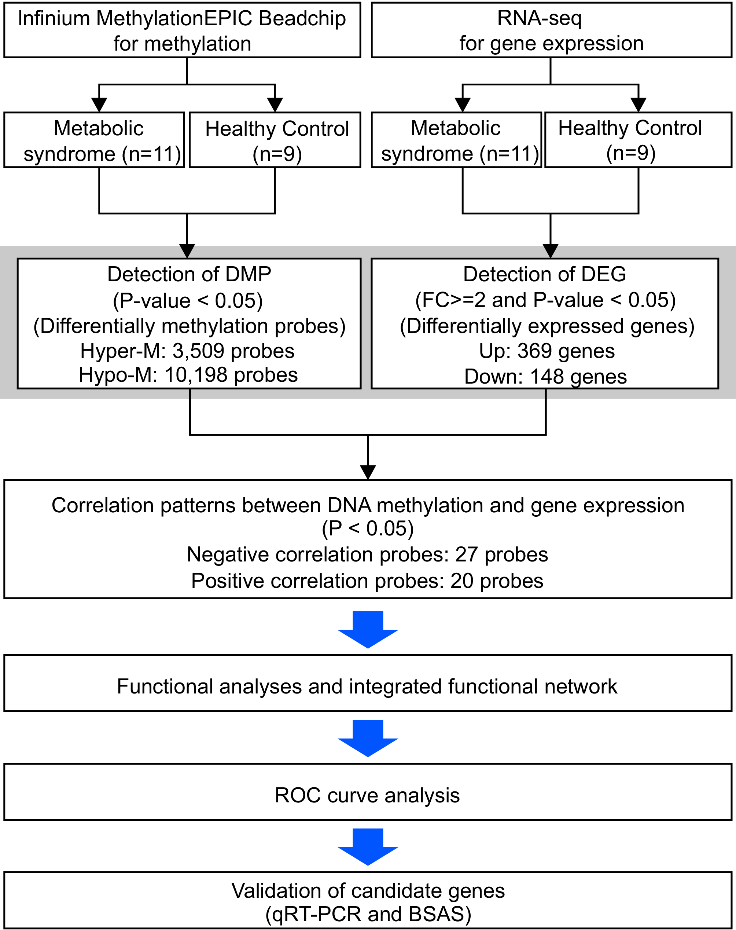


**Figure S1** The workflow used to identify metabolic syndrome (MetS)-associated genes regulated by DNA methylation.
