# Supplementary material for: Genome-wide DNA methylation profiling reveals candidate biomarkers and probable molecular mechanism of metabolic syndrome
Source: Genes Dis. 2022 Jan 11;9(4):833–6. doi: 10.1016/j.gendis.2021.12.010 (PMC9170599; doi:10.1016/j.gendis.2021.12.010)
Supplement: Multimedia component 2 [file mmc2.docx]

**Table S1. Demographics and characteristics of study population**

|  | | MetS (n=18) | Normal (n=14) | P |
| --- | --- | --- | --- | --- |
| All | Sex(men/women) | 11/7 | 5/9 |  |
|  | Age | 43.28 ± 7.50 | 39.57 ± 7.33 | 0.968 |
|  | BMI | 31.41 ± 4.54 | 24.04 ± 9.10 | 0.049 |
| Analysis set (n=20) | Sex(±men/women) | 7/4 | 1/8 |  |
|  | Age | 43.73 ± 8.73 | 42.82 ± 7.51 | 0.7 |
|  | BMI | 31.46 ± 5.09 | 24.57 ± 10.76 | 0.61 |
|  | *MetS risk factors* | | | |
|  | WC | 98.47 ± 9.07 | 80.68 ± 20.25 | 0.063 |
|  | Triglycerides | 179.55 ± 134.76 | 87.56 ± 37.71 | 0.068 |
|  | HDL cholesterol | 42.45 ± 7.59 | 55.56 ± 14.94 | 0.032 |
|  | SBP | 136.96 ± 18.07 | 110.67 ± 18.67 | 0.839 |
|  | DBP | 87.00 ± 19.45 | 67.33 ± 15.73 | 0.366 |
|  | Glucose | 100.09 ± 28.87 | 77.33 ± 7.58 | 0.043 |
| Validation set(n=12) | Sex (men/women) | 4/3 | 4/1 |  |
|  | Age | 43.86 ± 5.61 | 36.22 ± 5.94 | 0.65 |
|  | BMI | 31.31 ± 3.90 | 23.10 ± 6.00 | 0.51 |
|  | *MetS risk factors* | | | |
|  | WC | 99.48 ± 8.49 | 83.39 ± 16.44 | 0.18 |
|  | Triglycerides | 193.00 ± 49.17 | 148.60 ± 44.52 | 0.996 |
|  | HDL cholesterol | 44.86 ± 9.50 | 57.40 ± 22.50 | 0.01 |
|  | SBP | 139.07 ± 14.07 | 126.60 ± 11.08 | 0.882 |
|  | DBP | 91.57 ± 12.49 | 79.40 ± 9.13 | 0.43 |
|  | Glucose | 94.14 ± 27.15 | 84.20 ± 4.97 | 0.14 |

Data are represented by the least squares (LS) mean and standard error (SE)

*MetS: Metabolic syndrome, WC: waist circumference, SBP: systolic blood pressure, DBP: diastolic blood pressure

**Subjects with MetS:**

Diagnosis of MetS was based on the National Cholesterol Education Program-Adult Treatment Panel (NCEP-ATP) Ⅲ ^1^ criteria presented by the American Heart Association/National Heart, Lung, and Blood Institute. The diagnostic criteria for MetS risk factors included (1) high blood pressure (systolic blood pressure ≥ 130 mmHg, diastolic blood pressure ≥ 85 mmHg) or specific treatment, (2) fasting plasma glucose ≥ 100 mg/dL or specific treatment, (3) low HDL cholesterol (HDL-C < 40 mg/dL for men, HDL-C < 50 mg/dL for women) or specific treatment, (4) high triglyceride levels (150 mg/dL) or specific treatment, and (5) abdominal obesity with cutoffs specific to South Koreans (waist circumference ≥ 90 cm for men or ≥ 85 cm for women). MetS was defined when three or more of the aforementioned five risk factors were applicable.

**Reference**

1. Expert Panel on Detection E, Treatment of High Blood Cholesterol in A. Executive Summary of The Third Report of The National Cholesterol Education Program (NCEP) Expert Panel on Detection, Evaluation, And Treatment of High Blood Cholesterol In Adults (Adult Treatment Panel III). *JAMA*. May 16 2001;285(19):2486-97. doi:10.1001/jama.285.19.2486
