# Supplementary material for: Genome-wide DNA methylation profiling reveals candidate biomarkers and probable molecular mechanism of metabolic syndrome
Source: Genes Dis. 2022 Jan 11;9(4):833–6. doi: 10.1016/j.gendis.2021.12.010 (PMC9170599; doi:10.1016/j.gendis.2021.12.010)
Supplement: Multimedia component 7 [file mmc7.docx]

Table S6. List of significantly correlated genes between DNA methylation and gene expression

| **Gene** | **Probe** | **delta_beta** | **logFC** | **corr** | **P** |
| --- | --- | --- | --- | --- | --- |
| *TREML4* | cg03849834 | 0.1354381 | −5.12925 | −0.882 | 0 |
| *CD248* | cg06419846 | 0.0442044 | −1.00683 | −0.727 | 0 |
| *PDLIM4* | cg20512303 | −0.045101 | 1.5337333 | −0.708 | 0 |
| *SLC1A7* | cg01132471 | 0.0408064 | −1.295217 | −0.681 | 0.001 |
| *FLT4* | cg09050461 | −0.035452 | −1.163843 | 0.702 | 0.001 |
| *FLT4* | cg25935985 | −0.031087 | −1.163843 | 0.704 | 0.001 |
| *PDLIM4* | cg27615366 | −0.027818 | 1.5337333 | −0.647 | 0.002 |
| *MMEL1* | cg18932078 | −0.035656 | 1.0420869 | −0.643 | 0.002 |
| *GFPT2* | cg26523565 | −0.044207 | −2.434931 | 0.643 | 0.002 |
| *PDE10A* | cg08412584 | 0.0509037 | 2.3491424 | 0.644 | 0.002 |
| *CD248* | cg05496363 | 0.0352265 | −1.00683 | −0.636 | 0.003 |
| *PCDHB1* | cg06899976 | −0.024207 | 1.9693929 | −0.6 | 0.005 |
| *PDLIM4* | cg07262247 | −0.032843 | 1.5337333 | −0.597 | 0.005 |
| *NOS1AP* | cg26663636 | 0.0297289 | 1.4615302 | 0.588 | 0.006 |
| *GFPT2* | cg05904716 | −0.057567 | −2.434931 | 0.596 | 0.006 |
| *PTPRF* | cg05661060 | −0.013298 | 1.6437378 | −0.584 | 0.007 |
| *SPATA18* | cg03103192 | −0.04944 | 2.3431614 | −0.577 | 0.008 |
| *PEAR1* | cg17967261 | 0.0139054 | 1.408249 | 0.578 | 0.008 |
| *SLC8A3* | cg11802326 | 0.0110757 | 1.218463 | 0.567 | 0.009 |
| *PRDM6* | cg01085125 | 0.0148392 | −1.967823 | −0.563 | 0.01 |
| *MT1G* | cg01507019 | −0.036123 | 1.2553073 | −0.552 | 0.012 |
| *VEPH1* | cg01296532 | −0.009415 | 1.5685207 | −0.544 | 0.013 |
| *THRB* | cg07581492 | 0.0128371 | 1.182978 | 0.545 | 0.013 |
| *ESYT3* | cg01905210 | −0.041909 | −2.245994 | 0.536 | 0.015 |
| *HMGA2* | cg20088964 | −0.016726 | 1.218902 | −0.53 | 0.016 |
| *ASAP2* | cg17746316 | 0.0235788 | 1.0895021 | 0.518 | 0.019 |
| *COL13A1* | cg13680337 | −0.041965 | −1.263018 | 0.52 | 0.019 |
| *SHC2* | cg25264268 | −0.047669 | −1.658752 | 0.521 | 0.019 |
| *MYLK* | cg19534753 | −0.03011 | 1.0420038 | −0.513 | 0.021 |
| *LTBP1* | cg03906115 | 0.0650851 | 1.0761089 | 0.513 | 0.021 |
| *RAP1GAP* | cg24001246 | −0.036813 | −1.119922 | 0.508 | 0.022 |
| *GP6* | cg05374025 | −0.097345 | 1.0743536 | −0.496 | 0.026 |
| *GP6* | cg18355337 | −0.078968 | 1.0743536 | −0.487 | 0.03 |
| *GFPT2* | cg02891314 | 0.2530955 | −2.434931 | −0.482 | 0.031 |
| *MFSD2B* | cg12449528 | −0.020926 | 1.3588178 | −0.479 | 0.032 |
| *RHOBTB1* | cg15279476 | −0.033203 | 1.1035497 | −0.479 | 0.033 |
| *GP6* | cg05215830 | −0.054302 | 1.0743536 | −0.469 | 0.037 |
| *CYP19A1* | cg14694011 | 0.0198352 | 1.9319942 | 0.469 | 0.037 |
| *CMBL* | cg23284609 | −0.019961 | 1.027215 | −0.468 | 0.038 |
| *GFPT2* | cg23248424 | 0.2252503 | −2.434931 | −0.467 | 0.038 |
| *TTYH1* | cg00558215 | −0.027966 | −2.032532 | 0.466 | 0.038 |
| *DUOX1* | cg21183461 | −0.032489 | 1.3772493 | −0.464 | 0.039 |
| *SPATA18* | cg01020859 | −0.024085 | 2.3431614 | −0.463 | 0.04 |
| *MEGF11* | cg10461560 | −0.036614 | −1.89065 | 0.462 | 0.04 |
| *MFSD2B* | cg21129641 | −0.017269 | 1.3588178 | −0.46 | 0.041 |
| *PARS2* | cg10322308 | 0.0063296 | 1.0429674 | 0.45 | 0.047 |
| *SLC38A3* | cg19914607 | −0.015695 | −1.152161 | 0.448 | 0.048 |

* delta_beta: methylation difference, logFC: expression difference (log fold-change), corr: correlation coefficient
