# Supplementary material for: Genome-wide DNA methylation profiling reveals candidate biomarkers and probable molecular mechanism of metabolic syndrome
Source: Genes Dis. 2022 Jan 11;9(4):833–6. doi: 10.1016/j.gendis.2021.12.010 (PMC9170599; doi:10.1016/j.gendis.2021.12.010)
Supplement: Multimedia component 9 [file mmc9.docx]

Table S8. List of primer sequences used for GFPT2 qRT-PCR

| **Gene** | **Forward primer** | **Reverse primer** |
| --- | --- | --- |
| *GFPT2* | AGGATCCTTGCTTCGCCAAA | TGGGGCAGCTCAATTGTCTT |
| *GAPDH* | GAA GGT GAA GGT CGG AGT | GAA GAT GGT GAT GGG ATT TC |
