# Supplementary material for: Genome-wide DNA methylation profiling reveals candidate biomarkers and probable molecular mechanism of metabolic syndrome
Source: Genes Dis. 2022 Jan 11;9(4):833–6. doi: 10.1016/j.gendis.2021.12.010 (PMC9170599; doi:10.1016/j.gendis.2021.12.010)
Supplement: Multimedia component 10 [file mmc10.docx]

Table S9. List of primer sequences used for GFPT2 BSAS

| **Gene** | BS-primer_FWD (5' to 3') | BS-primer_REV (5' to 3') |
| --- | --- | --- |
| *GFPT2* | TTAAGAGGGGAGGGGAAAGGAAA | CACCTTAAATATAAAAATCAACC |

*Ref: hg19, Target position: chr5: 179740647-179741143 (+)
